# Cross-polarization coupling terahertz time-domain spectroscopy in a semiconductor based on the Hall effect

**DOI:** 10.1038/s41598-017-11055-w

**Published:** 2017-09-13

**Authors:** Jiangsheng Hu, JinSong Liu, Kejia Wang

**Affiliations:** Wuhan National Laboratory for Optoelectronics, School of Optical and Electronic Information, Huazhong University of Science and Technology, Wuhan, 430074 China

## Abstract

We propose a new type of terahertz time-domain spectroscopy in an isotropic semiconductor wafer applied by a magnetic field in which two cross-polarization THz pulses couple with each other via the Hall effect. We built a classic theoretic model to describe cross-polarization coupling THz spectroscopy (CPCTS). Numerical simulations show that the magnetic field can clearly affect the spectral features of the two THz pulses via the Hall effect in which both the magnitude and direction of the magnetic field and the thickness of the wafer play important roles. Using CPCTS, we present an improved method that is non-contact to measure the material parameters, such as the damping constant and carrier density of a semiconductor wafer, and discuss the possibility of THz functional devices. Finally, we describe an experimental scheme to guide CPCTS.

## Introduction

The Hall effect is a well-known physical phenomenon. Let us consider an isotropic semiconductor wafer, such as GaAs, described under a Cartesian coordinate (**e**_***x***_, **e**_***y***_, **e**_***z***_) system in which the *z*-axis is parallel to the normal of the wafer. In simple terms, if a static magnetic field and an electric field are applied along the **e**_***z***_ and **e**_***x***_ directions, an electric field will appear in the **e**_***y***_ direction. Terahertz (THz) radiation is such an electromagnetic wave with a vibration frequency that is far lower than that of infrared waves; thus, its electric field component is allowed to be static. Using the THz field to replace the applied electric field in the DC Hall effect, a quasi-Hall effect can occur, termed the “THz Hall effect”^1^. Combining the THz Hall effect THz time-domain spectroscopy (THz-TDS)^2^, measurements were performed tdetermine the carrier density and mobility^1^ and the Faraday rotation^3^ of a doped semiconductor wafer and to establish the complex conductivity tensor of superconductor thin films^4^. The THz Hall effect was also used to realize THz magnetospectroscopy in InAs-based heterostructures^5^ or in GaAs quantum wells^6^. The optical counterpart of the THz Hall effect is the optical Hall effect^7, 8^. By contrast, the THz Hall effect is more conceptually identical to the DC Hall effect. Combining THz technology with the optical Hall effect, a method for THz optical-Hall measurements was presented^9^ and used to measure the material parameters in a semiconductor^10^ and graphene^11^.

THz-TDS is an extremely useful spectroscopic tool, which generally involves one incoming THz beam. In this study, we propose a new type of THz-TDS in which two cross-polarization THz pulses are incident on an isotropic semiconductor wafer applied with a static magnetic field. For such a framework, natural questions to address are how the two pulses interact with each other in the wafer via the THz Hall effect and how the interaction affects the THz spectroscopy. To answer these questions, let us make a detailed analysis: Two THz pulses with interval *τ* are focused onto the face of a square wafer with its two sides along the **e**_***x***_ and **e**_***y***_ directions. The two THz fields are polarized along **e**_***x***_ and **e**_***y***_ and drive charge carriers drifting towards **e**_***x***_ and **e**_***y***_. In the presence of a magnetic field pointing parallel to the beam propagation direction, **e**_***z***_, the carriers drifting towards **e**_***x***_(**e**_***y***_) will form a Hall current oriented in the orthogonal direction, **e**_***y***_(**e**_***x***_), via a Lorentz force. The Hall current along **e**_***x***_(**e**_***y***_) will radiate a new THz field along **e**_***x***_(**e**_***y***_). As a result, a coupling between the two cross-polarization incoming THz beams will occur within the wafer via the THz Hall effect, which could support a new type of THz-TDS. Let us refer to this THz-TDS as cross-polarization coupling THz spectroscopy (CPCTS).

In this s, a classical theory was built to describe the CPCTS. The Hall effect is described b. yimple transverse magnetic field pattern via the Lorentz force. The motion of the carrier is described by the Drude model. The polarization induced by the THz fieis obtained by means of the electric dipole moment. The interaction between the THz fields in the wafer is described by a coupled propagation equation. By solving equation, the THz spectroscopy radiated from the wafer is numerically simulated. Our results indicate that there is an obvious coupling between the two cross-polarization THz fields and the spectrum profile of the output THz beam significantly changes compared with that of the incoming beam. Additionally, both the magnitude and direction of the magnetic field and the thickness of the wafer play important roles in these behaviours. In addition, we derive a set of analytical formulas to express the damping constant and the carrier density of a semiconductor as a function of the transmissivity spectrum that could be measured in a possible CPCTS experiment. In fact, these formulations provide a way to improve the non-contact measurements of the material parameters of the semiconductor wafer reported in ref. 1. We designed an experimental setup to make a detailed description of our vision, which is helpful to guide CPCTS experiments. Finally, certain ideas to design THz functional device are discussed. Our work constructs a bridge to connect THz-TDS with the THz Hall effect, which not only allows the building of a new type of THz-TDS but also provides new ideas for the design of THz devices.

## Classic Theory of CPCTS

**Figure 1:**
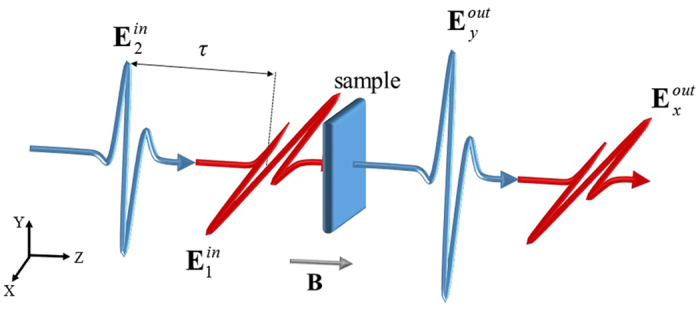
Framework of CPCTS. Two THz pulses (**E**_1_ and **E**_2_) are incident on an isotropic siconductor wafer along the *z* direction at time 0 and −*τ*. **E**_1_ and **E**_2_ are polarized along the *x* and *y* directions. **B** is the magnetic field applied on the wafer along the *z* direction.

Figure 1 shows the CPCTS framework. Two THz pulses (${{\bf{E}}}_{1}^{in}={E}_{x}{{\bf{e}}}_{x}$ and ${{\bf{E}}}_{2}^{in}={E}_{y}{{\bf{e}}}_{y}$) are incident on a semiconductor wafer at time 0 and −*τ*. The total incoming electric field is ${{\bf{E}}}^{in}(t,\tau )={E}_{x}(t){{\bf{e}}}_{x}+{E}_{y}(t-\tau ){{\bf{e}}}_{y}$, and *E*_*x*_ **e**_*x*_ and *E*_*y*_ **e**_*y*_ drive electrons drifting towards **e**_*x*_ and **e**_*y*_, respectively. When a magnetic field, **B** = *B* **e**_*z*_, is applied to the wafer, the motion of the electrons will be changed by the Lorentz force, **F** ∝ **v** × **B**, via the Hall effect, and here, **v** = v_*x*_ **e**_*x*_ + v_*y*_ **e**_*y*_ is the velocity vector of the electrons.

The electrons in the wafer are driven by the electric field forces from the two cross-polarization THz fields and the Lorentz force from the magnetic field via the Hall effect. The equations of motion of the electrons are described by the Drude model and are expressed in the *x*-axis and *y*-axis as the following.

1-1 $$\frac{{d}^{2}x}{d{t}^{2}}+{\gamma }_{0}\frac{dx}{dt}=\frac{1}{m}[{q}_{0}{E}_{x}(t)+{q}_{0}{v}_{y}B],$$

1-2 $$\frac{{d}^{2}y}{d{t}^{2}}+{\gamma }_{0}\frac{dy}{dt}=\frac{1}{m}[{q}_{0}{E}_{y}(t-\tau )-{q}_{0}{{\rm{v}}}_{x}B],$$

where ${\gamma }_{0}$, *m*, and ${q}_{0}$ are the damping constant, effective mass and charge of the electron, v_*x*_ = *dx*/*dt* and v_*y*_ = *dy*/*dt*. The magnetic field, *B*, results in the coupling of motion between the *x* and *y* directions via the Hall effect. By solving these equations in frequency domains, coordinates *x* and *y* can be obtained as follows.

2-1 $$x(\omega ,\tau )=\frac{{q}_{0}}{m}[{S}_{\alpha }(\omega ){E}_{x}(\omega )-{S}_{\beta }(\omega ){E}_{y}(\omega ){e}^{-i\omega \tau }],$$

2-2 $$y(\omega ,\tau )=\frac{{q}_{0}}{m}[{S}_{\beta }(\omega ){E}_{x}(\omega )+{S}_{\alpha }(\omega ){E}_{y}(\omega ){e}^{-i\omega \tau }],$$

where ${S}_{\alpha }(\omega )$ and ${S}_{\beta }(\omega )$ are the response functions, ${S}_{\alpha }(\omega )=(\alpha -\beta )/({\alpha }^{2}+{\beta }^{2})$, ${S}_{\beta }(\omega )=(\alpha +\beta )/({\alpha }^{2}+{\beta }^{2})$, $\alpha =i\omega {\omega }_{c}-i{\gamma }_{0}\omega -{\omega }^{2}$, $\,\beta =i\omega {\omega }_{c}+i{\gamma }_{0}\omega +{\omega }^{2}$, which is the cyclotron frequency.

From Eq. (2), the polarization vector can be obtained via the electric dipole moment:

3 $${\bf{P}}(\omega ,\tau )=N{q}_{0}[x(\omega ,\tau ){{\bf{e}}}_{x}+y(\omega ,\tau ){{\bf{e}}}_{y}],$$

where *N* is the density of the carrier.

The THz was in the medium satisfy the following one-dimensional propagation equation:

4 $$\frac{\partial }{\partial z}{\bf{E}}(z,t,\tau )=\varepsilon {\mu }_{0}\frac{{\partial }^{2}}{\partial {t}^{2}}{\bf{E}}(z,t,\tau )+{\mu }_{0}\frac{{\partial }^{2}}{\partial {t}^{2}}{\bf{P}}(z,t,\tau ).$$

By assuming a slowly varying envelope approximation, the envelope of the THz field in the frequency domain can be obtained as

5 $$2ik\frac{\partial {\bf{E}}(z,\omega ,\tau )}{\partial z}=-{\mu }_{0}{\omega }^{2}{\bf{P}}(z,\omega ,\tau ).$$

Substituting Eqs (2) and (3) into Eq. (5), the THz fields in the medium satisfy the following coupling equation

6 $$\frac{\partial }{\partial z}{\bf{E}}={\bf{AE}},$$

where $${\bf{E}}=(\begin{array}{c}{E}_{x}\\ {E}_{y}\end{array})$$, $${\bf{A}}=(\begin{array}{cc}\tilde{{S}_{\alpha }} & -\tilde{{S}_{\beta }}{e}^{-i\omega \tau }\\ \tilde{{S}_{\beta }} & \tilde{{S}_{\alpha }}{e}^{-i\omega \tau }\end{array})$$, $\,\tilde{{S}_{\alpha }}=a{S}_{\alpha }$, $\tilde{{S}_{\beta }}=a{S}_{\beta }$, and $\,a=i{\mu }_{0}{\omega }^{2}\frac{N{q}_{0}^{2}}{2km}$. The solution of Eq. (6) is $\,{\bf{E}}(z)={e}^{{\bf{A}}z}{\bf{E}}(0)=\sum _{j=0}^{\infty }\frac{{({\bf{A}}z)}^{j}}{j!}{\bf{E}}(0)$. Keeping the Taylor expansion in the first two terms (i.e., j = 0 and 1), the two orthogonal components of the THz field in the medium can be obtained:

7-1 $${E}_{x}(z,\omega ,\tau )=(1+\tilde{{S}_{\alpha }}z){E}_{x}(0,\omega )-\tilde{{S}_{\beta }}z{E}_{y}(0,\omega ){e}^{-i\omega \tau },$$

7-2 $${E}_{y}(z,\omega ,\tau )=\tilde{{S}_{\beta }}z{E}_{x}(0,\omega )+(1+\tilde{{S}_{\alpha }}z{e}^{-i\omega \tau }){E}_{y}(0,\omega ).$$

Letting *L* behe thickness of the wafer, the output THz fields from the wafer can be obtained by making *z* = *L* in Eq. (7), i.e., ${E}_{x}^{out}={E}_{x}(L,\omega ,\tau )$ and $\,{E}_{y}^{out}={E}_{y}(L,\omega ,\tau )$.

## Results and Discussion

**Figure 2:**
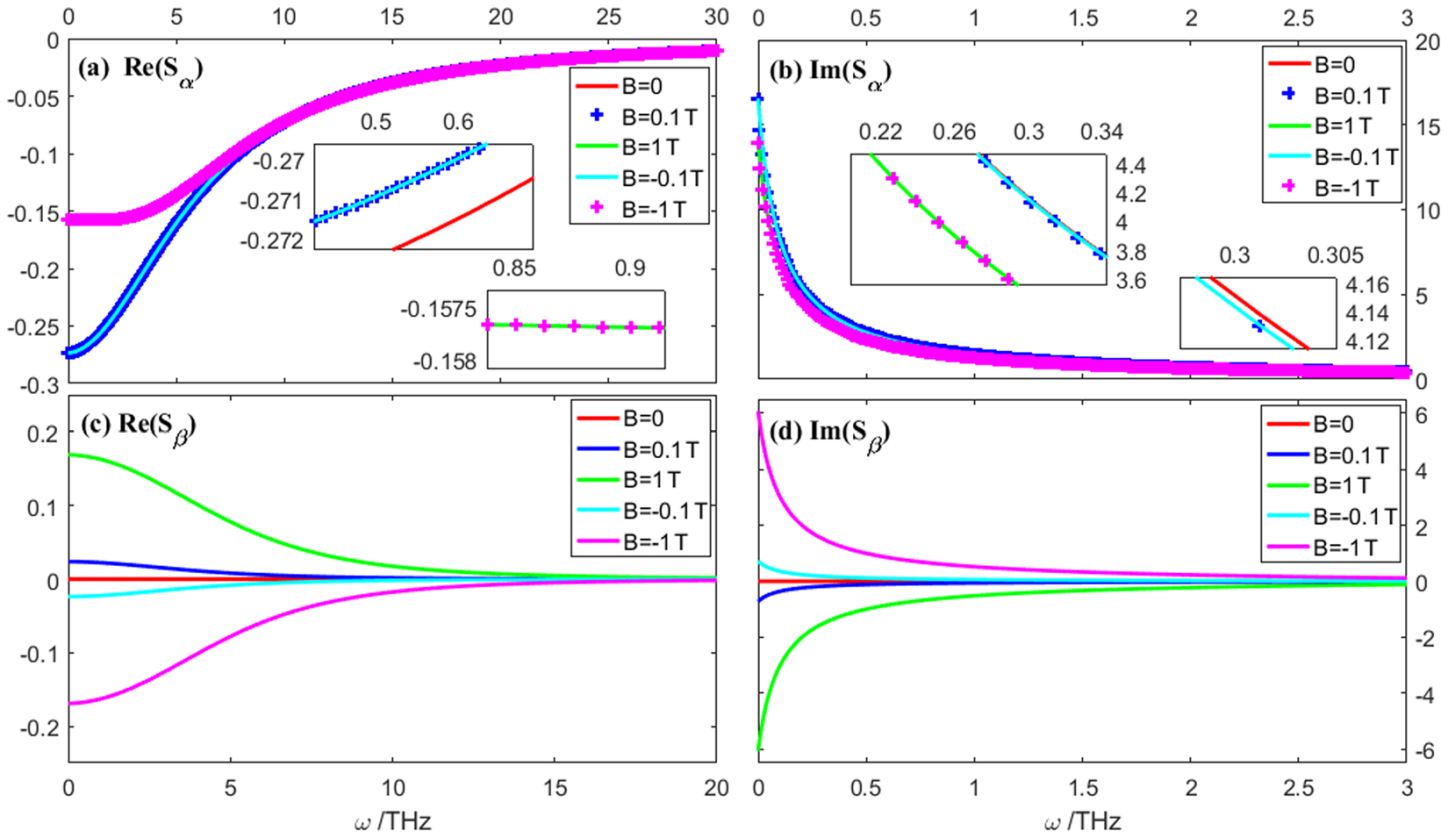
Curves of the response functions of *S*_*α*_(*ω*) and *S*_*β*_(*ω*) depending on the parameter for *B*. The model sample is a doped isotropic GaAs wafer with *γ*_0_ = 6.0288 THz, *q*_0_ = 1.602 × 10^−19^ C, *m*_e_ = 9.1^−31^ kg, and m = 0.067 *m*_*e*_. The insert figures are used to distinguish between these curves.

For the two response functions, *S*_*β*_ supports the cross-coupling, whereas *S*_*α*_ supports the response in the same polarization direction. *B* = 0 leads to *α* = −*β*, resulting in *S*_*β*_ ≠ 0. However, *S*_*α*_ ≠ 0, which means the cross-coupling cannot occur if *B* = 0, whereas the same polarization response can occur even if *B* = 0. *S*_*β*_ is nearly an odd function of *B*, which means that the direction of the applied magnetic field can have influence on the cross-coupling. Figure 2 shows the curves of *S*_*α*_(*ω*) and *S*_*β*_(*ω*), depending on the parameter for *B*. Note that *B* < 0 means that the magnetic field is applied along the −*z* direction. The magnetic field can obviously affect both the real and imaginary parts of *S*_*β*,_ and it can slightly affect those of *S*_α._ This effect is easily understood because *S*_α_, unlike *S*_β_, does not involve *B* in its numerator. These results indicate that the magnetic field, **B**, could obviously affect the CPCTS via the Hall effect.

The results above and Eq. (7) indicate that the two orthogonal components of the THz field in the medium are coupled with each other when the magnetic field **B** is applied. If **B** = 0, which leads to $\,\tilde{{S}_{\beta }}=0$, such a couple cannot occur. In this case, *E*_*x*_ and *E*_*y*_ will propagate independently in the medium. The magnitude and direction of **B**, the wafer thickness, *L*, and the THz pulses interval, *τ*, could have an effect on the coupling process. To reveal these influences, we will perform a numerical analysis using Eq. (7) in the case of ${|\tilde{{S}_{\alpha ,\beta }}L|}^{2}\ll |\tilde{{S}_{\alpha ,\beta }}L|\ll 1$ to ensure the Taylor series converges quickly such that using the Taylor expansion in the first two terms is sufficient. The temporal function of the incoming THz pulse is assumed to be $\,{E}_{x,y}^{in}(t)={E}_{0}sin({b}_{1}(t-{t}_{0})+{b}_{2}){e}^{-{(t-{t}_{0})}^{2}/2{\tau }_{0}^{2}}$ with *b*_1_ = 0.85*π*/*τ*_0_, *b*_2_ = 1.256, and ${\tau }_{0}=0.85$ ps. The other parameters used in the calculation are *m* = 0.067 *m*_*e*_ in which *m*_*e*_ is the electron mass, *q*_0_ is the elementary charge, and *μ*_0_ is the permeability in vacuum.

**Figure 3:**
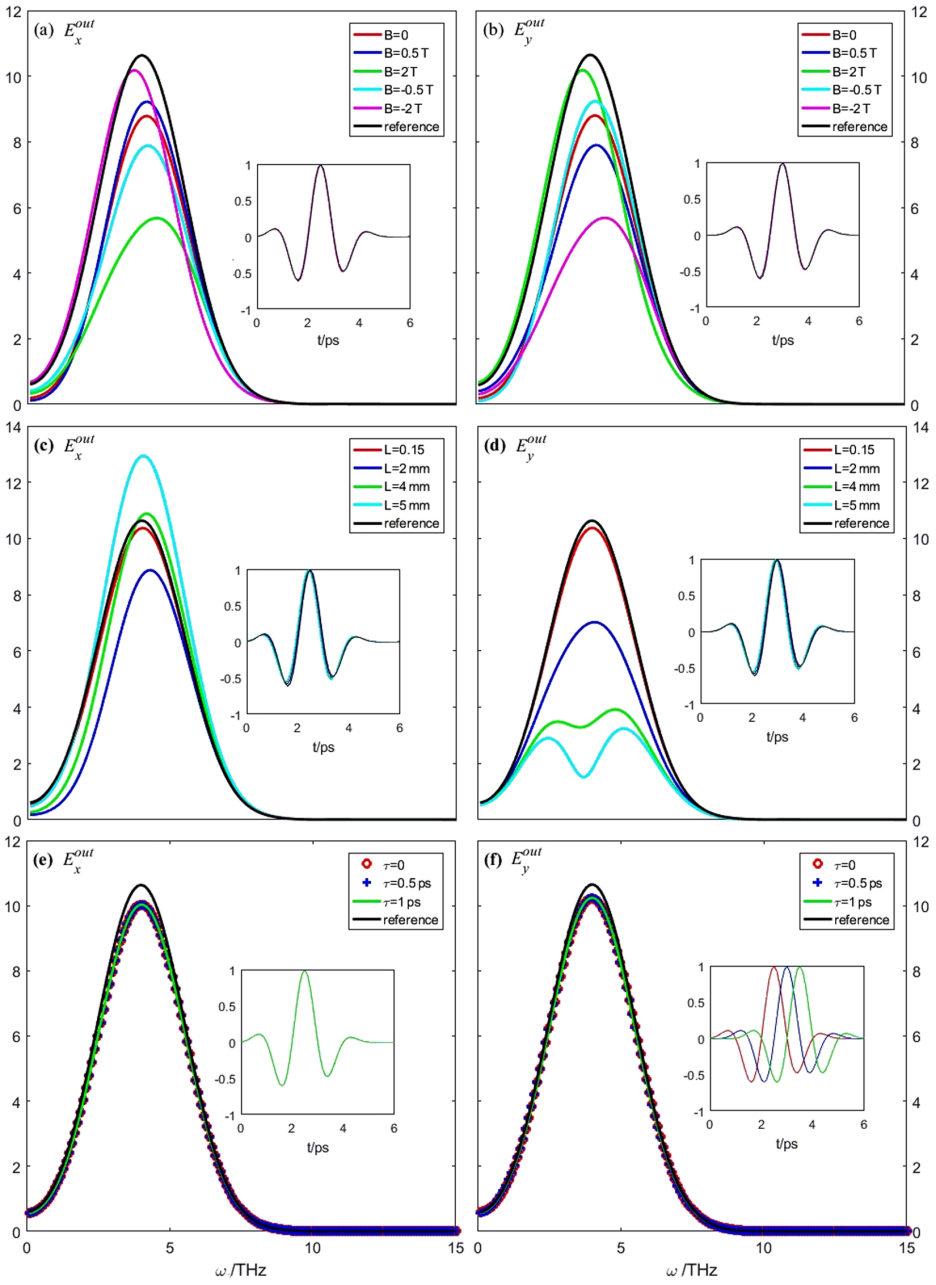
Curves of the frequency domain output of the THz fields, and the insert figures are the corresponding normalized time domain curves. The reference lines are the incoming THz fields,$\,{E}_{x,y}^{in}$, in frequency domain. (**a**) ${E}_{x}^{out}$ and (**b**) ${E}_{y}^{out}$ under $\tau =0.5\,{\rm{ps}}$, *L* = 2.0 mm, and *B* = 0, *B* ± 0.5 T, *B* = ±2.0 T; (**c**) ${E}_{x}^{out}$ and (**d**) ${E}_{y}^{out}$ unde*B* = 1.0 T, *τ* = 0.5 ps, and *L* = 0.15, 2.0, 4.0, 5.0 mm; (**e**) ${E}_{x}^{out}$ and (**f**) ${E}_{y}^{out}\,\,$unde*L* = 2.0 mm, *B* = 1.0 T, and *τ* = 0, 0.5, 1.0 ps.

**Figure 4:**
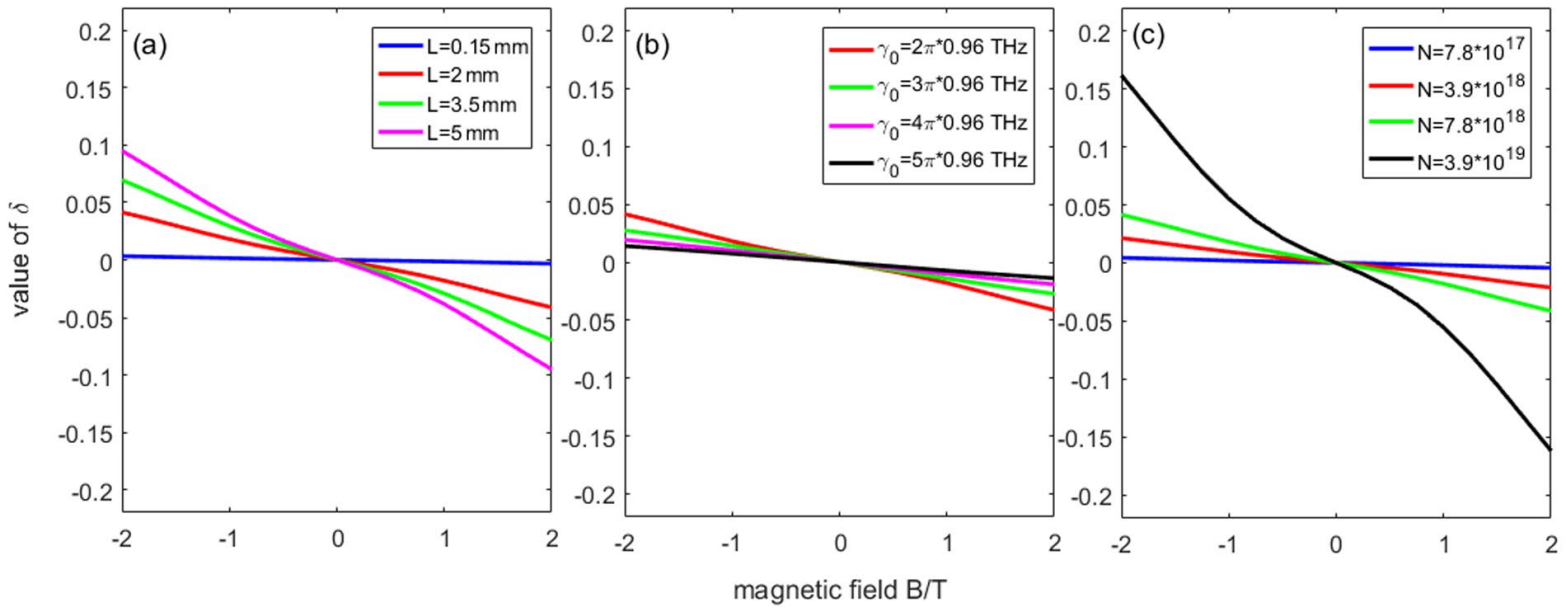
he dependences of (**a**) the wafer thickness *L*, (**b**) the damping constant *γ*_0_, and (**c**) the carrier density *N* on the coupling degree *δ*.

Figure 3 shows the curves of the output THz field, ${E}_{x}^{out}$ and ${E}_{y}^{out},$ both in the frequency and time domains. As shown in subfigures (a) and (b), when we keep *τ* = 0.5 ps and *L* = 2 mm, as the value of the static magnetic field increases, the coupling between the ${E}_{x}^{out}$ and ${E}_{y}^{out}$ obviously increases, and the direction of the magnetic field has an influence on the coupling. When B = 0, i.e., the magnetic field disappears, the *x*-component output field is the same as the y-component output field, which means there is no longer any coupling between them. Subfigures (c) and (d) show the dependence of the output THz field on the wafer thickness, *L*. Note that the reference line means *L* = 0; thus, as shown in the subfigure, there is no coupling between the two components of the output field. The coupling between ${E}_{x}^{out}$ and ${E}_{y}^{out}$ becomes more and more strong as *L* increases. When *L* is big enough, for example, *L* ≥ 4 mm, as shown in subfigures (c) and (d), most of the energy of the *y*-component field is coupled to the *x*-component field. Subfigures (e) and (f) show the dependence of the output THz field on the interval time, *τ*. As shown, in the range of *τ* = 0 to 1 ps, the influence of *τ* on the coupling is minimal. To analyse the coupling strength quantitatively, a parameter called the *coupling degree*, *δ*, is defined as

8 $$\delta =\frac{{P}_{x}^{out}-{P}_{y}^{out}}{{P}_{x}^{in}+{P}_{x}^{in}},$$

where $\,{P}_{x,y}^{in,out}={\int }_{0}^{\infty }{|{E}_{x,y}^{in,out}(\omega )|}^{2}d\omega$. The meaning of *δ* is the percentage of the energy transfer from one THz polarization component to the other. To reveal the effect of the material parameters on the coupling strength, the dependences of the wafer thickness, *L*, damping cons and the density of the carrier, *N*, on *δ* are calculated, as shown in Fig. 4. As shown, the larger the *L* or *N* is, the larger the *δ* is, whereas the larger the *γ*_0_ is, the smaller the *δ* is, indicating that the influence of the material parameters on the coupling strength is obvious.

The damping constant, *γ*_0_, and the carrier density, *N*, are two important material parameters useto frequently characterize doped semiconductors, and they determine many of the electrical and optical properties of the sample. THz-TDS with the THz Hall effect can provide a method to perform noncontact measurements of the two parameters. Letting ${T}_{x,y}={E}_{x,y}^{out}(\omega )/{E}_{x,y}^{in}(\omega )$ be the spectrum transmissivity, and relating the coefficients *T*_*x*_ and *T*_*y*_ to the desired quantities *N* and *γ*_0_ in two equations, the two parameters was calculated by use of the measured data of *T*_*x*,*y*_(*ω*) valid at any particular frequency within the bandwidth of the THz pulse^1^. A similar approach can be built using CPCTS. For the sake of simplicity, assuming $\,{E}_{x}^{in}(\omega )={E}_{y}^{in}(\omega )$, setting *τ* = 0, the expressions of *N* and *γ*_0_ are obtained in term of *T*_*x,y*_(*ω*) as

9 $$\,{\gamma }_{0}={\omega }_{c}{\rm{Re}}\{\frac{{T}_{x}+{T}_{y}-2}{{T}_{x}-{T}_{y}}\},\,N={\omega }_{c}\frac{2{n}_{0}m}{L{\mu }_{0}{q}_{0}^{2}C}{\rm{Re}}\{\frac{{(1-{T}_{x})}^{2}+{(1-{T}_{y})}^{2}}{{T}_{y}-{T}_{x}}\}$$

Comparing to the method in ref. 1, our approach does not need to introduce a propagation function *P*(*ω*) as did in ref. 1 because the propagation equation is used in our theoretical model. Therefore, Eq. (9) provides a way to improve the non-contact measurements of the material parameters of the semiconductor wafer reported in ref. 1.

## Designed Experimental Scheme

**Figure 5:**
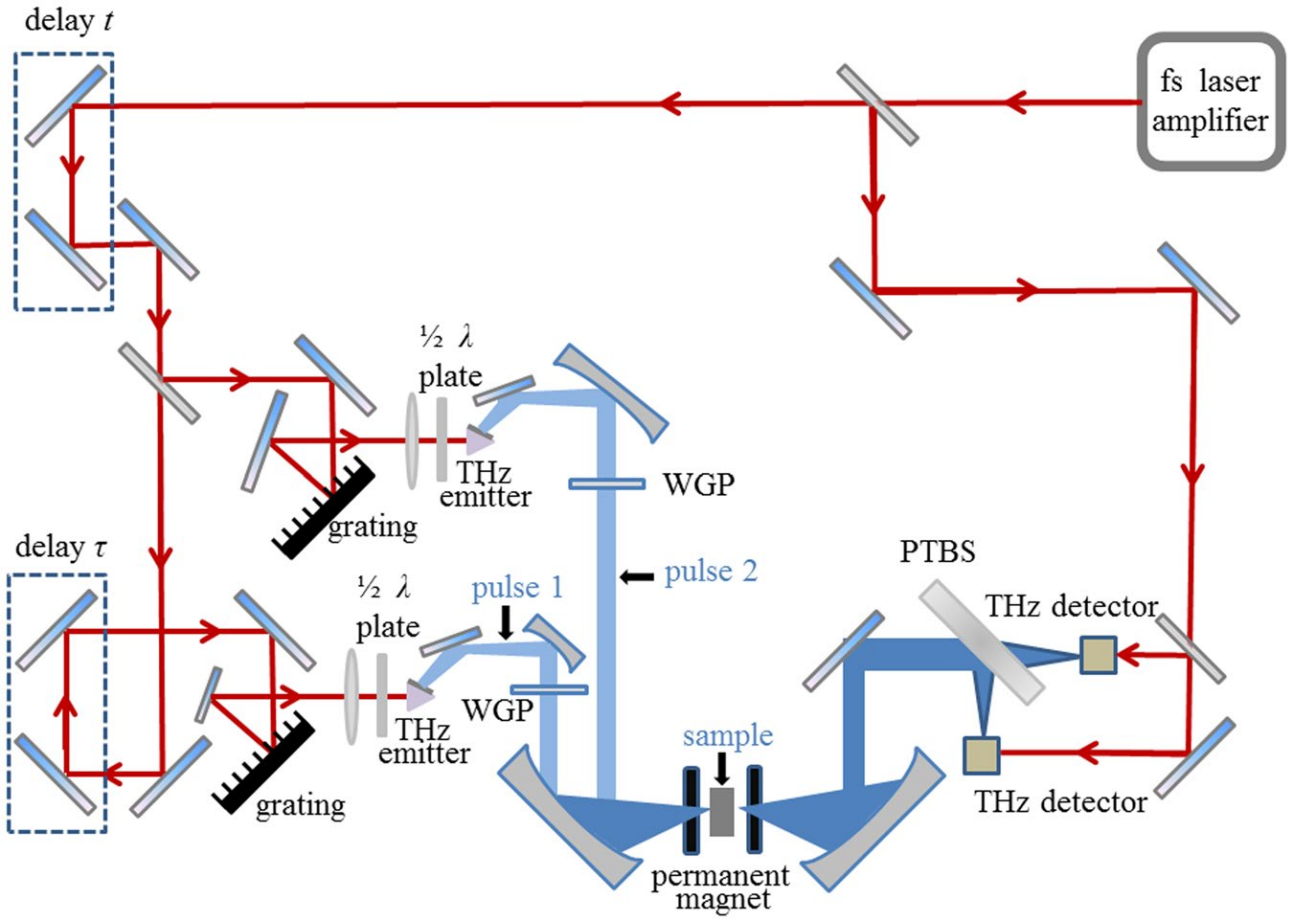
Scheme of the designed experiment for CPCTS. An fs laser beam will be split into two beams. Two THz pulses will be generated after the fs beams pass through the two LiNbO_3_ THz emitters, respectively. The polarization of the THz pulse will be adjusted by a wire gating polarizer (WGP). The angle between the two pulse’s polarizations will be set to 90°. The two THz pulses then will be focused and incident successively on a sample located in a permanent magnetic field. The output THz beam will be split into two orthogonal polarized beams by a polarizing THz beam splitter (PTBS), and the two output beams will be detected independently by two THz detectors. Delay *t* is the delay line existing in any traditional THz-TDS. Delay *τ* will be used to adjust the interval between the two THz pulses.

We designed a scheme to guide a CPCTS experiment as shown in Fig. 5, which can be regarded as an extension of the experiment in ref. 1. The main difference is that there are two cross-polarization THz incident pulses with interval, τ, in the designed scheme, thus allowing us to reveal the interaction between the two THz pulses with a time delay, which is not already accessible by previous approaches. In fact, regardless of the experimental setup in ref. 1 or the designed one that is just a variant of a traditional THz-TDS, for which each unit technology is mature and the challenge will arise from system integration, both require an experienced THz-TDS engineer to build the system and to perform the experiment.

By suitably adjusting the structure of CPCTS, we could design a number of THz functional devices, such as a THz modulator^12–14^ and a THz polarization rotator^15^. To be specific, in the framework of CPCTS, a THz modulator could be realized by putting an incident THz beam as the CW and the other as the DC square wave, whereas a THz polarization rotator could be realized by blocking one incoming THz beam.

## Conclusions

By adding the Hall effect to THz-TDS, we present a new type of THz time-domain spectroscopy: cross-polarization coupling THz spectroscopy. We build a classical theory to describe CPCTS. We theoretically reveal the interaction behaviour between two cross-polarization THz pulses in a semiconductor wafer applied with a static magnetic field via the THz Hall effect and the corresponding influence on the spectral features of the two THz pulses. In addition, we provide a way to improve non-contact measurements of the material parameters of the semiconductor wafer reported in ref. 1. Furthermore, we discuss the possibility of a THz modulator and a THz polarization rotator using CPCTS. We hope that our work will guide future CPCTS experiments and will stimulate even more fundamental developments in the new research field of THz spectroscopy.
